# Effectiveness of a Novel HIV Self-Testing Service with Online Real-Time Counseling Support (HIVST-Online) in Increasing HIV Testing Rate and Repeated HIV Testing among Men Who Have Sex with Men in Hong Kong: Results of a Pilot Implementation Project

**DOI:** 10.3390/ijerph18020729

**Published:** 2021-01-15

**Authors:** Paul Shing-fong Chan, Andrew Chidgey, Jason Lau, Mary Ip, Joseph T.F. Lau, Zixin Wang

**Affiliations:** 1 JC School of Public Health and Primary Care, Faculty of Medicine, The Chinese University of Hong Kong, Hong Kong, China; pchan@link.cuhk.edu.hk (P.S.-f.C.); mitk@cuhk.edu.hk (M.I.); 2 AIDS Concern, Hong Kong, China; andrew.chidgey@aidsconcern.org.hk (A.C.); jason.lau@aidsconcern.org.hk (J.L.)

**Keywords:** HIV self-testing, online real-time counseling, pilot implementation, outcome and process evaluation, men who have sex with men

## Abstract

HIV self-testing (HIVST) with online real-time counseling (HIVST-online) is an evidence-based intervention to increase HIV testing coverage and to ensure linkage to care for men who have sex with men (MSM). A community-based organization (CBO) recruited 122 MSM who had ever used HIVST-online (ever-users) and another 228 new-users from multiple sources and promoted HIVST-online. A free oral fluid-based HIVST kit was sent to all the participants by mail. Experienced HIVST administrators implemented HIVST-online by providing real-time instruction, standard-of-care pre-test and post-test counseling via live-chat application. The number of HIVST-online sessions performed was documented by the administrators. The post-test evaluation was conducted 6 months after the pre-test survey. At month 6, 63.1% of ever-users and 40.4% of new-users received HIVST-online. Taking other types of HIV testing into account, 79.4% of ever-users and 58.6% of new-users being followed up at month 6 received any HIV testing during the project period. Ever-users were more likely to receive HIVST-online and any HIV testing as compared to new-users. Four HIVST-online users were screened to be HIV positive and linked to the treatment. The process evaluation of HIVST-online was positive. Implementation of HIVST-online was helpful to improve HIV testing coverage and repeated HIV testing among Chinese MSM. A larger scale implementation should be considered.

## 1. Introduction

Human immunodeficiency virus (HIV) is a serious public health threat among men who have sex with men (MSM) [1,2,3]. In China, the National Health and Family Planning Commission estimated the overall HIV prevalence among MSM to be 7.7%, and over a quarter of new HIV cases were attributed to MSM [4]. In Hong Kong, the HIV prevalence among MSM was 6.54% in 2017 [5]. MSM accounted for 59.6% of all the reported new HIV cases in 2018 [6].

HIV testing plays a crucial role in the era of “treat all”. Antiretroviral therapy (ART) is highly effective in preventing HIV transmission (over 90%) under conditions of good adherence [7,8,9]. Therefore, the World Health Organization (WHO) recommends ART to all people living with HIV, regardless of their CD4 T cell level [10]. The high coverage of HIV testing among a high-risk population (e.g., MSM) is indispensable to achieve the 90–90–90 targets established by the Joint United Nations Program on HIV/AIDS (i.e., 90% of all people living with HIV know their HIV status, 90% of people with diagnosed HIV receive ART, and 90% of all people on an HIV treatment achieve viral suppression) [11]. With counseling in place, HIV testing and counseling can both detect HIV cases and reduce risk behaviors [12,13]. Therefore, international health authorities recommend MSM to take up HIV testing every 6 months [14,15].

The coverage of HIV testing among MSM was low in Hong Kong. It was estimated that only 80% of HIV-infected MSM were identified in Hong Kong [16]. Very few MSM performed repeated testing (taking up two episodes of HIV testing 6 months apart) as recommended [17]. HIV self-testing (HIVST) is a useful means to increase both the coverage and frequency of HIV testing among MSM [18]. The WHO strongly recommends that HIVST should be offered as an additional approach to existing HIV testing services [19]. At least 77 countries have adopted policies supporting HIVST [20]. In Hong Kong, HIVST kits are available in registered pharmaceutical stores or through online purchase. Starting from 2015, several community-based organizations (CBOs) started to provide HIVST services to MSM. In September 2019, the Hong Kong Department of Health also launched an HIVST program [21]. People can order HIVST kits and upload testing results via an online platform. Most of these existing services provided optional post-test counseling to users. Users can call through a hotline or submit their testing results online to obtain counseling services. 

The WHO recommends that pre-test and post-test counseling services should be provided during HIV testing services [22]. There is a concern that HIVST users may skip counseling services. Some researchers argued that the lack of counseling support is a key limitation of HIVST, which might delay linkage to care and treatment [23]. An innovative HIVST service model (HIVST-online) was developed for MSM in Hong Kong, which includes the online promotion of HIVST, delivery of free HIVST kits through mail/express, provision of online real-time instruction, and pre-test/post-test counseling for HIVST users via live chat application. In the pre-test counseling, the administrators assess users’ risk of HIV infection by asking several standard questions (e.g., use of condoms, alcohol/drug during sexual behaviors), inform users on their risk level and explain the rationale. For users at high risk, the administrators prepare them for potential positive testing results. For users without high risk, advices on maintaining safe sex practices are provided. In the end of pre-test counseling, the administrators seek users’ consent to perform HIVST. In the post-test counseling, the administrators interpret the testing results for the participants. For users receiving negative testing results, the administrators explain the window period, provide advices for safe sex practice, and emphasize the needs of regular testing. For users with positive testing results, the administrators provide mental health first aid, information on follow-up services, and facilitate them to receive confirmatory testing in the Department of Health [24]. A randomized controlled trial (RCT) showed that HIVST-online could significantly increase HIV testing uptake [24]. The United States Center for Disease Control and Prevention listed HIVST-online as an evidence-based intervention [25]. However, it is unclear whether HIVST-online is useful to increase repeated HIV testing among MSM.

A CBO having good access to local MSM adapted HIVST-online and started a pilot implementation in January 2017. We evaluated the effectiveness of HIVST-online in increasing HIV testing and repeated testing among MSM in Hong Kong. We compared the uptake of HIVST-online and any HIV testing during a 6-month follow-up period between MSM who were ever-users and new-users of HIVST-online. Indicators of the process evaluation included the level of satisfaction of HIVST-online implementation. In addition, baseline factors predicting the HIVST-online uptake were also investigated.

## 2. Materials and Methods 

### 2.1. Study Design

**Figure 1:**
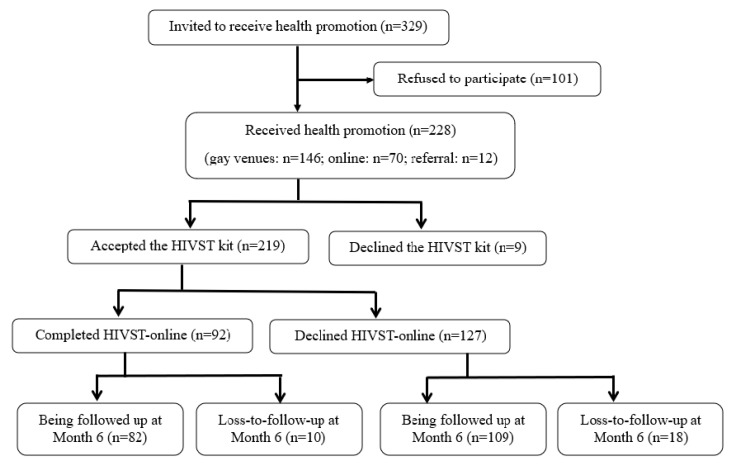
Flow diagram of the recruitment and uptake of human immunodeficiency virus self-testing (HIVST)-online among new-users.

**Figure 2:**
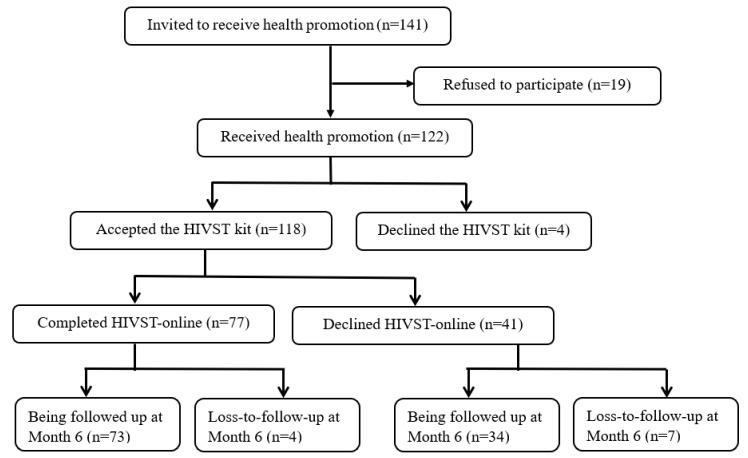
Flow diagram of the recruitment and uptake of HIVST-online among ever-users.

The pilot implementation was conducted from January to December 2017. All participants completed a baseline telephone survey before they received health promotion and implementation of HIVST-online, and completed another telephone survey 6 months after the baseline telephone survey. There was no control or comparison group. The study was registered at ClinicalTrials.gov, number NCT03268564. The flowchart diagram was shown in Figure 1 and Figure 2. 

### 2.2. Participants

Inclusion criteria were (1) Hong Kong Chinese speaking males aged ≥18 years old, (2) had anal sex with at least one man in the last 6 months, (3) willing to leave contacts to complete the post-test evaluation, and (4) having access to live-chat applications, e.g., Line (Line Corporation, Tokyo, Japan), Skype (Skype technologies, Microsoft, Palo Alto, CA, USA), or Facetime (Apple Inc., Cupertino, CA, USA). HIV-infected MSM were excluded.

### 2.3. Recruitment Process

The CBO staff approached MSM in six gay bars and six gay saunas at different time slots on weekdays and at weekends. The CBO staff also conducted online outreach by posting information on the study periodically as discussion topics on two gay websites with the highest traffic in Hong Kong. Interested participants could contact the CBO staff. Recruitment was supplemented by peer referrals. MSM experience a high level of stigma in China and the pre-test/post-test measurements included sensitive topics such as sexual behaviors and history of sexually transmitted infections. The signature on the informed consent document would be the only record linking the participant to the research and the principal risk of harm to the subject would be a breach of confidentiality. Therefore, we sought the endorsement from the Institutional Review Board (IRB) to waive participants’ signature on informed consent documents. Verbal informed consent was obtained according to the IRB guideline [26]. Participants were briefed on the research procedures and before the administration of the data collection, informed that they had the right to terminate the study at any time, and were given an information sheet (either a hardcopy on site or a softcopy through online communication) explaining the purpose, procedures, potential psychological distress caused by HIVST, benefits of joining the program, and their right to end participation at any time. The CBO staff signed a form pledging that the participants had been fully informed about the program. Participants were also briefed on data security. The pledge forms and their contact information were kept separately and stored in a locked cabinet. The participants’ name or contact information would not appear on the questionnaire or in the dataset. Multiple forms of contact information were obtained in order to make an appointment to conduct the pre-test survey and health promotion. Out of 329 MSM who were eligible to join the program, 101 (30.7%) refused to participate since they did not have time and/or for other logistical reasons; 228 (67.3%) participated in the program and received health promotion (gay venues: *n* = 146; online: *n* = 70; referral: *n* = 12) (Figure 1).

In addition, the CBO staff also approached HIVST-online ever-users in the previous RCT [24]. The procedures to obtain informed consent were similar to those for new-users. There were 189 MSM who had used HIVST-online in the previous RCT, the CBO staff was able to contact 141 of them, 19 refused to participate since they did not have time, and 122 provided verbal informed consent to join the program (Figure 2). Therefore, the pilot program covered 350 MSM in Hong Kong, including 228 new-users and 122 ever-users.

### 2.4. Training of CBO Staff

Eight CBO staff received 14-h basic motivational interviewing (MI) training by an experienced trainer who is a member of the Motivational Interviewing Network of Trainers. They worked as fieldworkers for health promotion. They were trained to deliver MI over the phone to increase their confidence. The role-plays and protocol specific practice were conducted twice a week for 4 weeks, and maintained by coaching sessions every 2 weeks by the same trainer. Fieldworkers were not deployed in the fieldwork unless they achieved an adequate level of competence. Ongoing support and supervision was provided by the trainer. In addition, six CBO staff received a 4-h training workshop on HIVST-online. They worked as HIVST-online administrators in the pilot program. 

### 2.5. Baseline Telephone Survey and Health Promotion

By appointment and through telephone, the CBO staff conducted the 10-min baseline survey. All participants were then sent a link to access the project webpage. The project webpage contained the following health promotion components during the project period:

(1) An online video promoting HIVST-online: The video was used in the previous RCT [24]. In the video, a local MSM narratively discussed the benefits and barriers of HIVST-online, demonstrated its procedures, and emphasized that HIVST was easy to use, as well as the availability of immediate online support.
(2) A new online video promoting regular HIV testing. The video was based on the maintenance theories [27]. In the video, a peer MSM shared his positive experience of using HIVST-online, and emphasized the importance of regular HIV testing.
(3) An online demonstration video on how to use oral fluid-based HIVST kits.
(4) Other health promotion components, including: (i) description of the project, (ii) knowledge and benefit of the HIVST, (iii) information about HIV epidemic among MSM in Hong Kong, (iv) a discussion forum containing positive feedbacks of HIVST-online users, and (v) contact of the program staff (phone, social media, and email).

In addition, new-users received a 15-min MI session over the phone. Fieldworkers made an appointment with the participants to conduct the MI. Ever-users did not receive MI, as they were already motivated to receive the service.

### 2.6. Implementation of HIVST-Online

The implementation was similar to that of the RCT [24].

(1) Receiving a free oral fluid-based HIVST kit: The HIVST kit used in this study (AwareTM Oral) is the brand of the Calypte Biomedical Corporation in the United States. It has a proven sensitivity/specificity of >99.8% and has been approved for marketing by the State of Food and Drug Administration in China. The kit was available online at a price of about HKD 200 (USD 25.8) during the project period. Unless there is an objection, all the participants were sent a free HIVST kit in a plain envelope without any identification of the program. Participants could also receive the kit sent by express to an address or pick it up in person by mail or express.

(2) Prospective users made appointments with HIVST-online administrators by phone/social media after they have received the HIVST kit.

(3) Through video chat, the administrators explained how to use the HIVST and sent the aforementioned demonstration video to the users if needed. They guaranteed that participants were anonymous and did not need to show their faces if desired and that no taping would be made. 

(4) The standard-of-care pre-test counseling was also provided, which covered the knowledge of HIV prevention, risk assessment, and benefits of HIV testing.

(5) Participants performed HIVST, under online and real-time supervision provided by the administrators. It took about 20 min to know the screening results. Users showed the test results visually to the administrators. The standard-of-care post-test counseling was then provided (15–25 min), which covered the following topics: (i) Explanation of the testing results, (ii) reminders for those who received negative results about their risk of HIV infection, and assistance for setting up specific goals for safe-sex behaviors, and (iii) psychological support for those who received positive results, which emphasized that they had to take up a free confirmatory HIV antibody testing offered by the Department of Health. Referrals would be made whenever needed. They could visit the CBO, and CBO staff would accompany them to visit the Department of Health if desired. The entire HIVST-online process took about 60 min, which was comparable to that needed for HIV testing and counseling in CBO or governmental clinics.

### 2.7. Month 6 Follow-Up Telephone Survey

Participants were invited to complete a follow-up telephone survey 6 months after the baseline survey. Up to five calls were made at different timeslots during weekdays/weekends before considering a case as loss-to-follow-up. A total of 191 (83.8%) new-users and 107 (87.7%) ever-users completed the follow-up survey.

### 2.8. Measurements

#### 2.8.1. Primary Outcome

The primary outcome is whether the participant has taken up HIVST-online between the baseline and follow-up surveys during a 6-month follow-up period. This outcome was recorded by HIVST-online administrators. At month 6, participants were also asked whether they received other forms of HIV testing during the follow-up period. 

#### 2.8.2. Baseline Background Characteristics 

Information collected included socio-demographics, sexual orientation, utilization of HIV/STI prevention services, history of HIV and other STIs, smoking, and drinking. Queried sexual behaviors included anal intercourse with regular and non-regular sex partners, condomless anal intercourse (CAI) with men, multiple male sex partnerships, use of sexual potency drugs, and engagement in sexualized drug use in the past 6 months. Regular male sex partners (RP) were defined as lovers and/or stable boyfriends, while non-regular partners (NRP) were defined as causal sex partners and/or male sex workers. In this study, sexualized drug use was defined as the use of any psychoactive substance before or during sexual intercourse [28].

#### 2.8.3. Perceptions Related to HIVST Measured at the Baseline

Six scales were constructed based on the Health Belief Model (HBM) [29]. They were: (1) The two-item Perceived Logistical Benefit Scale, (2) the three-item Perceived Psychological Benefit Scale, (3) the four-item Perceived Logistical Barrier Scale, (4) the four-item Perceived Psychological Barrier Scale, (5) the two-item Cue to Action Scale, and (6) the four-item Perceived Self-Efficacy Scale. The response categories for these scales were 1 = strongly disagree, 2 = disagree, 3 = neutral, 4 = agree, and 5 = strongly agree. The Cronbach’s alpha of these scales ranged from 0.64 to 0.89, single factors were identified by the exploratory factor analysis, explaining 48.3–89.9% of the total variance. 

In addition, two single items measured the behavioral intention to take up HIVST-online in the next 6 months (response categories: 1 = unlikely, 2 = neutral, 3 = likely) and perceived importance of real-time counseling supporting HIVST users (1 = very unimportant, 2 = unimportant, 3 = neutral, 4 = important, 5 = very important). 

#### 2.8.4. Process Evaluation

The fieldworkers recorded the starting/ending time of the MI as verification. The HIVST administrator kept a log sheet for each user to ensure key steps of the pre-test and post-test counseling were completed. 

The process evaluation of health promotion and HIVST-online implementation was conducted at month 6. Participants were asked: (1) Whether the content of the online video was clear, (2) whether the content of the online video was attractive, (3) whether they were satisfied with the project webpage, and (4) whether the health promotion had increased their understanding on the importance of regular HIV testing and their willingness to take up HIV testing. New-users were asked an additional question about their satisfaction of the MI session. HIVST-online users were asked about the satisfaction of the logistics of implementation and performance of the HIVST-online administrators. 

### 2.9. Ethics Statement

All subjects gave their informed consent for inclusion before they participated in the study. The study was approved by the Survey and Behavioral Research Ethics Committee, the Chinese University of Hong Kong (reference: KPF16ICF10).

### 2.10. Statistical Analysis

The difference in baseline characteristics between new-users and ever-users, and between those who declined and who received HIVST-online at month 6 were compared using X^2^ tests or independent sample *t*-tests. Logistic regression models were used to test the between-group differences in the uptake of HIVST-online and any HIV testing, after controlling any baseline variables that showed *p* < 0.05 in between-group comparisons. Crude odds ratios (OR) and adjusted odds ratios (AOR) were obtained. Baseline characteristics of participants who completed the month 6 post-test survey and those who were lost-to-follow-up were also compared. Using the HIVST-online uptake during the follow-up period as the dependent variable, and baseline background characteristics as an independent variable, OR predicting that the dependent variable were obtained using logistic regression models. After adjustment for those variables with *p* < 0.05 in the univariate analysis, the association between perceptions related to HIVST and the dependent variable were then assessed by AOR. Each AOR was obtained by fitting a single logistic regression model, which involved one of the perceptions and the significant background variables. SPSS version 21.0 (Chicago, IL, USA) was used for data analysis and *p*-values < 0.05 were considered as statistically significant.

## 3. Results

### 3.1. Baseline Characteristics

Over half of the participants were aged 18–30 years (57.1%), currently single (83.1%), had attended at least a college education (86.6%), full-time employed (84.3%), and identified themselves as gay (93.1%). In the past 3 months, 70% and 45.1% had anal intercourse with RP and NRP. The prevalence of multiple male sex partnerships, CAI with men, and sexualized drug use was 43.7%, 35.1%, and 3.4% in the past 3 months, respectively. Regarding the history of HIV testing, 38.6% received more than three episodes of HIV testing in addition to HIVST-online in the past 3 years. Item responses and scale scores of perceptions related to HIVST were shown in Table 1.

As compared to ever-users of HIVST-online, new-users were more likely to be cohabited/married with a man (*p* = 0.01), with a history of STI (*p* = 0.004), had received more than three episodes of HIV testing in addition to HIVST-online (*p* = 0.002), reported anal intercourse with RP in the past 3 months (*p* = 0.02), and scored higher in the Perceived Logistical Benefit Scale (*p* = 0.01), the Perceived Psychological Benefit Scale (*p* < 0.001), and the Perceived Psychological Barrier Scale (*p* < 0.001).

When comparing those who were followed up and were lost to follow-up, a significant difference was found in perceived logistical benefits among new-users (*p* = 0.02) and perceived importance of real-time counseling service supporting HIVST among ever-users (*p* = 0.01) (Table S1).

### 3.2. HIV Testing Uptake during the Follow-Up Period

As documented by the HIVST administrators, 40.4% (92/228) of new-users and 63.1% (77/122) of ever-users received HIVST-online during the project period. Among the participants who were followed up at month 6, 58.6% (112/191) and 79.4% (85/107) used any HIV testing during the follow-up period. As compared to new-users, ever-users were more likely to receive HIVST-online (AOR: 3.01, 95% CI: 1.80, 5.05) and any HIV testing (AOR: 4.82, 95% CI: 2.51–9.28) (Table 2).

Four HIVST-online users were screened to be HIV positive. Facilitated by the administrators, all of them received confirmatory HIV testing in the Department of Health and confirmed to be HIV positive. All of them received the appropriate treatment and care services.

### 3.3. Factors Predicting HIVST-Online Uptake

Being ever-users of HIVST-online at the baseline was associated with higher uptake of HIVST-online during the follow-up period (OR: 2.53, 95% CI: 1.61, 3.98) in the univariate analysis (Table 3). The behavioral intention to take up HIVST-online at the baseline was also associated with higher uptake of HIVST-online in the univariate analysis (OR: 1.67, 95% CI: 1.03, 2.72) but not in the adjusted analysis (Table 4).

### 3.4. Process Evaluation of HIVST-Online Users

As recorded by the fieldworkers, the duration of MI ranged from 11 to 19 min. All HIVST-online administrators closely followed the protocol. Among the HIVST-online users who completed the process evaluation (*n* = 125), 88.8–96.8% were satisfied/very satisfied with different procedures of HIVST-online, 72.0–97.6% believed that the online real-time counseling was helpful in different aspects such as understanding their current risk, testing results, concept of window period, and reducing their fear toward HIV testing and high-risk behaviors (Table 5).

### 3.5. Baseline Characteristics between Those Who Declined and Received HIVST-Online

When comparing those who declined HIVST-online to those who received HIVST-online, a significant difference was found in the behavioral intention to use the free HIVST-online with real-time counseling services in the coming 6 months (*p* = 0.02). No significant difference was found in the other characteristics (Table S2). 

## 4. Discussion

Our results showed that implementing HIVST-online is helpful in increasing HIV testing coverage among MSM in Hong Kong. The proportion of MSM who had taken up any type of HIV testing in our project (66.1%) was higher than that of a multimedia campaign promoting HIV testing in Hong Kong (43.1%) [30]. The prevalence of HIVST uptake in our project (48.3%) was comparable to a successful social entrepreneurship HIVST service model in Southern China (46.8%) [31]. There is also room to improve the effectiveness of the health promotion for HIVST-online. The current health promotion for HIVST-online was standard and one-off. The meta-analysis showed that interventions tailored to one’s stage of change are more effective than non-stage-tailored, especially among less-motivated individuals (e.g., new-users) [32]. In addition, studies showed that people might move forward to the later SOC, go backward to the earlier SOC, or stay in the same SOC after being exposed to health promotion [33]. Therefore, it is highly recommended that stage-tailored interventions should have multiple sessions and each session should tailor to the people’s current SOC [34]. Future programs should explore the effectiveness of applying multiple sessions of stage-tailored interventions to promote HIVST-online. In addition, about 20% of new-users and ever-users used other forms of HIV testing rather than HIVST-online. Future programs should also consider linking MSM to other forms of HIV testing at the same time (e.g., HIV testing at CBO), as some of them may not prefer HIVST-online.

It is encouraging to observe that about 80% of HIVST-online ever-users received any HIV testing during the project period, and the majority of them used HIVST-online again. Therefore, implementation of HIVST-online is of good potential to increase the regular HIV testing rate, which is very low among Chinese MSM [17]. Regular HIV testing is important for MSM as their sexual risk behaviors are continuous.

According to the process evaluation results shown in Table 5, 65.5–98.3% of new-users and 77.6–97.0% of ever-testers of HIVST-online believed the counseling service supporting HIVST users to be important. HIVST-online users were satisfied with the real-time counseling. With real-time counseling in place, administrators know all the users’ sero-status and provide immediate support to the users. This is especially important to facilitate those who received positive HIVST results to receive confirmatory testing, treatment, and care. In our project, all the users who received positive HIVST results received immediate support and were linked to care and treatment. In contrast, only 55% of HIVST service users would seek a confirmatory test in a clinic if they got an HIV-positive test in previous studies [35]. Ensure linkage to care is one major strength of HIVST-online. 

Based on the findings presented in Table 3 and Table 4, being ever-users and having a behavioral intention to use HIVST at the baseline predicted the HIVST-online uptake during the project period, while the associations between HIVST-online uptakes and other background variables or baseline perceptions were non-significant. Such non-significant findings also had some implications. MSM with different socio-demographic, behavioral characteristics, and perceptions responded positively and similarly to the HIVST-online promotion. 

This study has the strengths of a low drop-out rate, validated primary outcome, and good process evaluation. However, it also has several limitations. First, there was no control group, since HIVST-online had already been evaluated by RCT. The aim of this study was to evaluate its effectiveness in real-world settings rather than its efficacy. Second, the uptake of other types of HIV testing was self-reported and was likely to be over-reported due to social desirability. Third, a selection bias might exist, as we did not collect information of the MSM who refused to join the study. They may have different characteristics as compared to the participants. Fourth, an attrition bias might also exist. However, this should be small as the drop-out rate is low. Finally, this study used convenience sampling, since probability sampling was not feasible. In addition, a selection bias existed. Therefore, cautions should be taken when generalizing the results to the MSM in Hong Kong or other Chinese cities.

## 5. Conclusions

The implementation of HIVST-online was helpful in increasing the HIV testing rate among MSM and ensure good linkage to care among HIVST users in Hong Kong. It is also of good potential to increase regular HIV testing. A larger scale implementation should be considered.

## Figures and Tables

**Table 1:** Background characteristics of the participants.

	All (*n* = 350)	New-Users of HIVST-Online (*n* = 228)	Ever-Users of HIVST-Online (*n* = 122)	*p*-Value
	%	%	%	
Socio-demographic characteristics				
Age group (years)				
18–30	57.1	54.4	62.3	
31–40	31.4	34.6	25.4	
>40	11.4	11.0	12.3	0.21
Marital/cohabitation status				
Currently single	83.1	79.4	90.2	
Cohabitate/married with a man	16.6	20.6	9.0	
Cohabited/married with a woman	0.3	0	0.8	0.01
Highest education level attained				
Secondary or below	13.4	12.7	14.8	
College or above	86.6	87.3	85.2	0.60
Current employment status				
Full-time	84.3	86.0	81.1	
Part-time/unemployed/retired/student	15.7	14.0	18.9	0.24
Sexual orientation				
Gay	93.1	93.4	92.6	
Bisexual	6.3	5.7	7.4	
Heterosexual	0.6	0.9	0	0.49
History of sexually transmitted infection				
No	79.1	74.1	87.7	
Yes	20.9	25.4	12.3	0.004
HIV testing history				
No. of HIV testing in the past 3 years in addition to HIVST-online				
0	17.4	16.2	19.7	
1–3	44.0	38.6	54.1	
>3	38.6	45.2	26.2	0.002
Sexual behaviors in the last 3 months				
Anal intercourse with regular male sex partner(s) (RP)				
No	30.0	25.9	36.9	
Yes	70.0	74.1	62.3	0.02
Anal intercourse with non-regular male sex partner(s) (NRP)				
No	54.6	57.0	50.0	
Yes	45.1	43.0	49.2	0.24
Condomless anal intercourse (CAI) with men				
No	54.9	57.0	50.8	
Yes	35.1	34.6	36.1	0.52
Multiple male sex partnerships				
No	56.3	59.2	50.8	
Yes	43.7	40.8	49.2	0.13
Illicit drug use before/during anal intercourse with men				
No	96.6	96.5	96.7	
Yes	3.4	3.5	3.3	0.91
Perceptions related to HIV testing				
Behavioral intention to use free HIVST with real-time counseling services in the coming 6 months				
Unlikely/neutral	25.7	26.3	24.6	
Likely	74.3	73.7	75.4	0.73
Perceived logistical benefits of HIVST (% agree/strongly agree)				
HIVST is easy for you to use	76.0	74.1	79.5	
HIVST is convenient for you	82.3	81.6	83.6	
Perceived Logistical Benefit Scale ^1^ (Mean/SD)	8.1/1.6	7.9/1.5	8.4/1.8	0.01
Perceived psychological benefits of HIVST (% agree/strongly agree)				
Using HIVST could reduce embarrassment	79.1	77.6	82.0	
Using HIVST could avoid being stigmatized by service providers	50.0	42.5	63.9	
Using HIVST could protect your privacy	84.0	82.0	87.7	
Perceived Psychological Benefit Scale ^2^ (Mean/SD)	11.5/2.6	10.9/2.6	12.4/2.4	<0.001
Perceived logistical barriers of HIVST (% agree/strongly agree)				
HIVST is expensive for you	57.1	59.6	52.5	
It is difficult for you to buy a HIVST kit	54.0	51.8	58.2	
You do not know how to choose a reliable HIVST kit	70.0	69.3	71.3	
You are concerned about the accuracy of HIVST	53.4	57.9	45.1	
Perceived Logistical Barrier Scale ^3^ (Mean/SD)	14.0/3.2	13.9/3.2	14.2/3.2	0.33
Perceived psychological barrier of HIVST (% agree/strongly agree)				
You are not psychologically prepared to perform HIVST	19.4	17.1	23.8	
You are concerned about not understanding the HIVST results	13.1	13.2	13.1	
You cannot receive immediate psychological support if you have a positive HIVST result	40.3	35.1	50.0	
You cannot access the HIV treatment and care services if you have a positive HIVST result	27.1	24.6	32.0	
Perceived Psychological Barrier Scale ^4^ (Mean/SD)	10.6/3.1	10.0/3.1	11.6/2.7	<0.001
Cue to action related to HIVST (% agree/strongly agree)				
Significant others will support you to do HIVST	70.3	68.4	73.8	
Male sex partner will support you to do HIVST	78.9	79.4	77.9	
Cue to Action Scale ^5^ (Mean/SD)	7.9/1.6	7.8/1.5	8.1/1.9	0.05
Perceived self-efficacy related to HIVST (% agree/strongly agree)				
You are confident to obtain a high-quality HIVST kit	38.0	39.9	34.4	
You are confident to use HIVST kits properly	74.0	73.7	74.6	
You are confident to understand the HIVST results	78.6	79.4	77.0	
You are confident to receive confirmatory testing after obtaining a positive HIVST result	78.3	80.7	73.8	
Perceived Self-efficacy Scale ^6^ (Mean/SD)	14.8/2.4	14.8/2.4	14.9/2.4	0.63
Perceived importance of real-time counseling service supporting HIVST users				
Very unimportant/ unimportant/neutral	36.0	38.6	31.1	
Important/very important	64.0	61.4	68.9	0.17

*p*-values were obtained using the X^2^ test (for categorical variables) or independent-sample *t*-tests (for continuous variables); ^1^ Perceived Logistical Benefit Scale, two items, Cronbach’s alpha = 0.89, one factor was identified by the exploratory factor analysis, explaining 89.9% of total variance; ^2^ Perceived Psychological Benefit Scale, three items, Cronbach’s alpha = 0.72, one factor was identified by the exploratory factor analysis, explaining 66% of total variance; ^3^ Perceived Logistical Barrier Scale, four items, Cronbach’s alpha = 0.67, one factor was identified by the exploratory factor analysis, explaining 51.4% of total variance; ^4^ Perceived Psychological Barrier Scale, four items, Cronbach’s alpha = 0.64, one factor was identified by the exploratory factor analysis, explaining 48.3% of total variance; ^5^ Cue to Action Scale, two items, Cronbach’s alpha = 0.84, one factor was identified by the exploratory factor analysis, explaining 86.6% of total variance; ^6^ Perceived Self-efficacy Scale, four items, Cronbach’s alpha = 0.67, one factor was identified by the exploratory factor analysis, explaining 53.4% of total variance.

**Table 2:** Types of HIV testing taken up by the participants during the follow-up period.

	All	New-Users of HIVST-Online	Ever-Users of HIVST-Online	Ever-Users vs. New-Users			
	%	%	%				
Among All Participants (*n* = 350)	*n* = 350	*n* = 228	*n* = 122	OR (95%CI)	*p*-Value	AOR (95%CI)	*p*-Value
HIVST-online uptake as documented by the HIVST-administrators (% Yes)	48.3	40.4	63.1	2.53 (1.61–3.98)	<0.001	3.01 (1.80–5.05)	<0.001
Among participants who completed the month 6 post-test survey (*n* = 298)	*n* = 298	*n* = 191	*n* = 107	OR (95%CI)	*p*-value	AOR (95%CI)	*p*-value
Uptake of other types of HIV testing reported by the participants at the post-test survey (% Yes)							
Using HIVST kits obtained in the project without receiving online real-time counseling services	1.0	0.5	1.9	3.62 (0.32–40.39)	0.30	13.99 (0.86–226.91)	0.06
Self-purchased HIVST kits and used by themselves	0.7	1.0	0	N.A.	N.A.	N.A.	N.A.
HIV testing at governmental hospitals or clinics	5.0	4.7	5.6	1.20 (0.42–3.47)	0.74	1.54 (0.46–5.19)	0.48
HIV testing at non-governmental organizations (NGO)	21.1	20.9	21.5	1.03 (0.58–1.84)	0.91	2.43 (1.16–5.07)	0.02
HIV testing at private clinics/laboratories	1.3	0.5	2.8	5.48 (0.56–53.36)	0.14	4.47 (0.40–50.16)	0.23
Any type of HIV testing	66.1	58.6	79.4	2.73 (1.57–4.73)	<0.001	4.82 (2.51–9.28)	<0.001

OR: Crude odds ratios; AOR: Adjusted odds ratios, odds ratios adjusted for baseline variables with *p* < *0*.05 in between-group comparisons in Table 1.

**Table 3:** Baseline background variables predicting the uptake of HIVST-online (*n* = 350).

	OR (95% CI)	*p*-Value
Socio-demographic characteristics		
Age group (years)		
18–30	1.0	
31–40	1.31 (0.84–2.12)	0.23
>40	0.85 (0.43–1.69)	0.64
Marital/cohabitation status		
Currently single	1.0	
Cohabitate/married with a man	0.60 (0.34–1.07)	0.08
Cohabited/married with a woman	N.A.	N.A.
Highest education level attained		
Secondary or below	1.0	
College or above	0.80 (0.43–1.48)	0.47
Current employment status		
Full-time	1.0	
Part-time/unemployed/retired/student	0.67 (0.37–1.21)	0.18
Sexual orientation		
Gay	1.0	
Bisexual	0.48 (0.19–1.20)	0.12
Heterosexual	1.03 (0.06–16.52)	0.99
History of sexually transmitted infection		
No	1.0	
Yes	0.79 (0.47–1.33)	0.38
HIV testing history		
Number of HIV testing in the past 3 years		
0	1.0	
1–3	0.99 (0.55–1.80)	0.99
>3	1.07 (0.58–1.96)	0.82
Being new-users or ever-users of HIVST-online		
New-users	1.0	
Ever-users	2.53 (1.61–3.98)	<0.001
Sexual behaviors in the last 3 months		
Anal intercourse with regular male sex partner(s) (RP)		
No	1.0	
Yes	0.71 (0.45–1.12)	0.14
Anal intercourse with non-regular male sex partner(s) (NRP)		
No	1.0	
Yes	1.42 (0.93–2.16)	0.11
Condomless anal intercourse (CAI) with men		
No	1.0	
Yes	1.28 (0.82–2.02)	0.28
Multiple male sex partnerships		
No	1.0	
Yes	1.27 (0.83–1.94)	0.27
Illicit drug use before/during anal intercourse with men (sexualized drug use)		
No	1.0	
Yes	1.52 (0.47–4.89)	0.48

OR: Crude odds ratios.

**Table 4:** Factors predicting the uptake of HIVST-online (*n* = 350).

	OR (95% CI)	*p*-Value	AOR (95% CI)	*p*-Value
Perceived Logistical Benefit Scale	1.00 (0.88–1.14)	0.95	0.98 (0.84–1.15)	0.82
Perceived Psychological Benefit Scale	1.01 (0.93–1.09)	0.91	0.99 (0.91–1.09)	0.91
Perceived Logistical Barrier Scale	1.03 (0.96–1.10)	0.39	1.03 (0.96–1.11)	0.39
Perceived Psychological Barrier Scale	1.00 (0.93–1.07)	0.96	0.99 (0.91–1.07)	0.74
Cue to Action Scale	0.98 (0.86–1.12)	0.78	0.98 (0.84–1.13)	0.74
Perceived Self-efficacy Scale	1.01 (0.92–1.10)	0.91	1.01 (0.91–1.11)	0.89
Perceived importance of real-time counseling supporting HIVST users				
Very unimportant/unimportant/neutral	1.0		1.0	
Important /very important	1.48 (0.95–2.30)	0.08	1.40 (0.88–2.21)	0.15
Behavioral Intention to take up free HIVST in the coming 6 months				
Unlikely/neutral	1.0		1.0	
Likely	1.67 (1.03–2.72)	0.04	1.60 (0.95–2.69)	0.08

OR: Crude odds ratios; AOR: Adjusted odds ratios, odds ratios adjusted for being new-users or ever-users of HIVST-online.

**Table 5:** Process evaluation of HIVST-online users (among those being followed up at month 6, *n* = 125).

	All (*n* = 125)	New-Users of HIVST-online (*n* = 58)	Ever-Users of HIVST-online (*n* = 67)	*p*-Value
	%	%	%	
Level of satisfaction of different procedures of HIVST-online (% satisfied/very satisfied)				
Receiving HIVST kits	92.0	89.7	94.0	0.37
Making appointment of HIVST-online	96.0	98.3	94.0	0.23
Visual and sound quality of online counseling	92.8	96.6	89.6	0.13
Clarity of instruction	96.8	98.3	95.5	0.38
Professionalism of HIVST-administrators	96.8	100.0	94.0	0.06
Credibility of HIVST-administrators	96.8	98.3	95.5	0.38
Support from HIVST-online administrators	92.8	96.6	89.6	0.13
Recommendations made by HIVST-online administrators on reducing high-risk behaviors	88.8	89.7	88.1	0.99
How helpful is online real-time counseling in the following aspects				
Understanding your current risk of HIV infection	85.6	82.8	88.1	0.40
Reducing your fear of HIV testing	72.0	65.5	77.6	0.13
Preparing you to do HIV testing	78.4	72.2	83.6	0.13
Mastering the methods and procedures of HIVST	96.0	94.8	97.0	0.53
Understanding testing results	97.6	98.3	97.0	0.65
Understanding the concept of window period	88.8	87.9	89.6	0.77
Acquiring knowledge to prevent HIV infection	85.6	87.9	83.6	0.49
Reducing high-risk behaviors	80.0	81.0	79.1	0.79
Behavioral intention to use HIVST-online again				
Very unlikely/unlikely/neutral	21.6	24.1	19.4	
Likely/very likely	78.4	75.9	80.6	0.52
Behavioral intention to recommend MSM friends to use HIVST-online				
Very unlikely/unlikely/neutral	23.2	27.6	19.4	
Likely/very likely	76.8	72.4	80.6	0.28

*p*-value was obtained by using the χ^2^ test.

## Data Availability

Data are contained within the article or supplementary materials.

## Supplementary Materials

The following are available online at https://www.mdpi.com/1660-4601/18/2/729/s1. Table S1: Comparing baseline characteristics of participants who completed the follow-up evaluation at month 6 versus those who were lost at follow-up; Table S2. Comparing baseline characteristics of participants who declined HIVST-online and completed HIVST-online at Month 6 follow-up.
