# Supplementary material for: Effectiveness of a Novel HIV Self-Testing Service with Online Real-Time Counseling Support (HIVST-Online) in Increasing HIV Testing Rate and Repeated HIV Testing among Men Who Have Sex with Men in Hong Kong: Results of a Pilot Implementation Project
Source: Int J Environ Res Public Health. 2021 Jan 15;18(2):729. doi: 10.3390/ijerph18020729 (PMC7830557; doi:10.3390/ijerph18020729)
Supplement: Supplementary file 1 [file ijerph-18-00729-s001.pdf]

Table S1. Comparing baseline characteristics of participants who completed the follow-up evaluation at Month 6 versus those who were lost to follow-up.

	**New-users of HIVST-online**			**Ever-users of HIVST-online**		
	**Being followed up at Month 6 (n=191)**	**Loss-to-follow-up (n=37)**	**P value**	**Being followed up at Month 6 (n=107)**	**Loss-to-follow-up (n=15)**	**P value**
	%	%		%	%	
**Socio-demographic characteristics**						
Age group						
18-30	56.0	45.9		58.9	86.7	
31-40	33.0	43.2		27.1	13.3	
>40	11.0	10.8	.47	14.0	0	.10
Marital/cohabitation status						
Currently single	80.6	73.0		89.7	93.3	
Cohabitate/married with a man	19.4	27.0		9.3	6.7	
Cohabitate/married with a woman	0	0	.29	0.9	0	.88
Education level						
Secondary or below	12.0	16.2		15.9	6.7	
University or above	88.0	83.8	.49	84.1	93.3	.35
Employment status						
Full-time	87.4	78.4		82.2	73.3	
Part-time/ unemployed/retired/student	12.6	21.6	.15	17.8	26.7	.41
Sexual orientation						
Gay	93.7	91.9		92.5	93.3	
Bisexual	5.2	8.1		7.5	6.7	
Heterosexual	1.0	0	.66	0	0	.91
History of sexually transmitted infection						
No	73.3	78.4		87.9	86.7	
Yes	26.2	21.6	.55	12.1	13.3	.90
**HIV testing history**						

Number of HIV testing in the past three years in addition to HIVST-online						
0	16.2	16.2		18.7	26.7	
1-3	36.6	48.6		56.1	40.0	
>3	47.1	35.1	.34	25.2	33.3	.50
**Sexual behaviors in the last three months**						
Anal intercourse with regular male sex partner(s) (RP)						
No	26.7	21.6		37.4	33.3	
Yes	73.3	78.4	.52	61.7	66.7	.74
Anal intercourse with non-regular male sex partner(s) (NRP)						
No	54.5	70.3		49.5	53.3	
Yes	45.5	29.7	,08	49.5	46.7	.81
Condomless anal intercourse (CAI) with men						
No	57.6	54.1		52.3	40.0	
Yes	34.6	35.1	.84	33.6	53.3	.20
Multiple male sex partnerships						
No	56.5	73.0		50.5	53.3	
Yes	43.5	27.0	.06	49.5	46.7	.84
Illicit drug use before/during anal intercourse with men (sexualized drug use)						
No	96.9	94.6		97.2	93.3	
Yes	3.1	5.4	.49	2.8	6.7	.43
**Perceptions related to HIV testing**						
Behavioral intention to use free HIVST with real-time counseling services in the coming six months						
Unlikely/neutral	26.2	27.0		22.4	40.0	
Likely	73.8	73.0	.92	77.6	60.0	.14
**Perceived logistical benefits of HIVST (% agree/strongly agree)**						
HIVST is easy for you to use	76.3	62.2		83.2	53.3	
HIVST is convenient for you	82.7	75.7		86.0	66.7	
Perceived Logistical Benefit Scale (Mean/SD)	8.0/1.4	7.4/1.7	.02	8.5/1.8	7.5/1.8	.05

**Perceived psychological benefits of HIVST (% agree/strongly agree)**						
Using HIVST could reduce embarrassment	77.5	78.4		83.2	73.3	
Using HIVST could avoid you being stigmatized by service providers	42.4	43.2		63.6	66.7	
Using HIVST could protect your privacy	81.2	86.5		87.9	86.7	
Perceived Psychological Benefit Scale (Mean/SD)	11.0/2.6	10.9/2.5	.84	12.4/2.4	12.2/2.1	.71
**Perceived logistical barriers of HIVST (% agree/strongly agree)**						
HIVST is expensive for you	62.8	43.2		51.4	60.0	
It is difficult for you to buy a HIVST kit	51.8	51.4		60.7	40.0	
You do not know how to choose a reliable HIVST kit	68.6	73.0		72.0	66.7	
You concern about the accuracy of HIVST	57.6	59.5		45.8	40.0	
Perceived Logistical Barrier Scale (Mean/SD)	13.9/3.1	13.8/3.5	.80	14.3/3.2	13.9/2.9	.64
**Perceived psychological barrier of HIVST (% agree/strongly agree)**						
You are not psychologically prepared to perform HIVST	10.5	24.3		23.4	26.7	
You concern about not understanding the HIVST results	13.6	10.8		12.1	20.0	
You cannot receive immediate psychological support if you have a positive HIVST result	36.1	29.7		50.5	46.7	
You cannot access HIV treatment and care services if you have a positive HIVST result	26.2	43.2		31.8	33.3	
Perceived Psychological Barrier Scale (Mean/SD)	9.9/3.2	10.4/2.8	.42	11.6/2.6	12.3/3.1	.34
**Cue to action related to HIVST (% agree/strongly agree)**						
Significant others will support you to do HIVST	71.2	54.1		73.8	73.3	
Male sex partner will support you to do HIVST	81.2	70.3		77.6	80.0	
Cue to Action Scale (Mean/SD)	7.8/1.5	7.5/1.6	.20	8.1/1.9	8.1/1.4	.99
**Perceived self-efficacy related to HIVST (% agree/strongly agree)**						

You are confident to obtain a high-quality HIVST kit	42.4	27.0		31.8	53.3	
You are confident to use HIVST kits properly	74.9	67.6		76.6	66.7	
You are confident to understand the HIVST results	80.1	75.7		76.6	80.0	
You are confident to receive confirmatory testing after obtaining a positive HIVST result	80.6	81.1		73.8	73.3	
Perceived Self-efficacy Scale (Mean/SD)	14.9/2.4	14.1/2.7	.06	14.9/2.4	15.1/2.8	.71
**Perceived importance of real-time counseling service supporting HIVST users**						
Very unimportant/ unimportant/neutral	38.7	37.8		27.1	60.0	
Important/very important	61.3	62.2	.92	72.9	40.0	.01

Table S2. Comparing baseline characteristics of participants who declined HIVST-online and completed HIVST-online at Month 6 follow-up.

	Those who declined HIVST-Online (n = 143)	Those who completed HIVST-Online (*n* = 155)	*p*-Value
	%	%	
Socio-demographic characteristics			
Age group (years)			
18–30	58.7	55.5	
31–40	28.0	33.5	
>40	13.3	11.0	0.54
Marital/cohabitation status			
Currently single	81.1	86.5	
Cohabitate/married with a man	18.2	13.5	
Cohabited/married with a woman	0.7	0	0.24
Highest education level attained			
Secondary or below	11.2	15.5	
College or above	88.8	84.5	0.28
Current employment status			
Full-time	84.6	86.5	
Part-time/unemployed/retired/student	15.4	13.5	0.65
Sexual orientation			
Gay	90.9	95.5	
Bisexual	8.4	3.9	
Heterosexual	0.7	0.6	0.21
History of sexually transmitted infection			
No	76.8	80.6	
Yes	23.2	19.4	0.41
HIV testing history			
No. of HIV testing in past 3 years in addition to HIVST-online			
0	16.8	17.5	
1–3	44.8	42.9	
>3	38.5	39.6	0.95
Sexual behaviors in the last three months			
Anal intercourse with regular male sex partner(s) (RP)			
No	28.7	32.9	
Yes	71.3	67.1	0.43
Anal intercourse with non-regular male sex partner(s) (NRP)			
No	56.6	49.0	
Yes	42.7	51.0	0.43
Condomless anal intercourse (CAI) with men			
No	56.6	54.8	
Yes	32.2	36.1	0.56
Multiple male sex partnerships			
No	57.3	51.6	
Yes	42.7	48.4	0.32
Illicit drug use before/during anal intercourse with men			
No	97.9	96.1	
Yes	2.1	3.9	0.50
Perceptions related to HIV testing			

Behavioral intention to use free HIVST with real-time counseling services in the coming six months			
Unlikely/neutral	30.8	19.4	
Likely	69.2	80.6	0.02
Perceived logistical benefits of HIVST (% agree/strongly agree)			
HIVST is easy for you to use	82.4	76.1	
HIVST is convenient for you	84.6	83.2	
Perceived Logistical Benefit Scale [1] (Mean/SD)	8.2/1.5	8.2/1.7	0.70
Perceived psychological benefits of HIVST (% agree/strongly agree)			
Using HIVST could reduce embarrassment	79.7	79.4	
Using HIVST could avoid being stigmatized by service providers	50.3	49.7	
Using HIVST could protect your privacy	83.9	83.2	
Perceived Psychological Benefit Scale [2] (Mean/SD)	11.5/2.6	11.5/2.7	0.98
Perceived logistical barriers of HIVST (% agree/strongly agree)			
HIVST is expensive for you	60.8	56.8	
It is difficult for you to buy a HIVST kit	52.4	57.4	
You do not know how to choose a reliable HIVST kit	67.1	72.3	
You concern about the accuracy of HIVST	49.0	57.4	
Perceived Logistical Barrier Scale [3] (Mean/SD)	13.9/3.3	14.1/3.0	.59
Perceived psychological barrier of HIVST (% agree/ strongly agree)			
You are not psychologically prepared to perform HIVST	18.2	18.8	
You concern about not understanding the HIVST results	13.3	12.9	
You cannot receive immediate psychological support if you have a positive HIVST result	40.6	41.9	
You cannot access HIV treatment and care services if you have positive HIVST result	28.2	28.4	
Perceived Psychological Barrier Scale [4] (Mean/SD)	10.5/3.1	10.5/3.0	0.87
Cue to action related to HIVST (% agree/strongly agree)			
Significant others will support you to do HIVST	74.1	70.3	
Male sex partner will support you to do HIVST	82.4	78.1	
Cue to Action Scale [5] (Mean/SD)	8.0/1.6	7.9/1.7	0.78
Perceived self-efficacy related to HIVST (% agree/strongly agree)			
You are confident to obtain a high-quality HIVST kit	42.0	35.5	
You are confident to use HIVST kits properly	74.1	76.1	

You are confident to understand the HIVST results	79.7	78.1	
You are confident to receive confirmatory testing after obtaining a positive HIVST result	79.0	77.4	
Perceived Self-efficacy Scale [6] (Mean/SD)	14.9/2.5	15.0/2.2	0.70
Perceived importance of real-time counseling service supporting HIVST users			
Very unimportant/ unimportant/neutral	39.2	30.3	
Important/very important	60.8	69.7	0.12
